# VdPRMT1 Is Required for Fungal Growth, Metabolism, and Pathogenicity in *Verticillium dahliae*

**DOI:** 10.3390/cells15151425

**Published:** 2026-08-06

**Authors:** Wenwen Li, Suoxian Li, Siyuan Wu, Xi Jin, Huiming Guo, Hongmei Cheng, Yue Li, Wenfang Guo, Xiaofeng Su

**Affiliations:** 1 School of Agriculture and Biomanufacturing, Zhengzhou University, Zhengzhou 450001, China; 2 Key Laboratory of Development and Application of Rural Renewable Energy, Biogas Institute of Ministry of Agriculture and Rural Affairs, Chengdu 610041, China; 3 National Key Laboratory of Agricultural Microbiology, Biotechnology Research Institute, Chinese Academy of Agricultural Sciences, Beijing 100081, China; 4 Baoding University, Baoding 071000, China; 5 Key Laboratory of Biological Ecological Adaptation and Evolution in Extreme Environments, College of Life Science, Xinjiang Agricultural University, Urumqi 830052, China

**Keywords:** *Verticillium dahliae*, protein arginine methyltransferase (PRMT), fungal pathogenicity, carbon metabolism, cell wall integrity, protein–protein interaction

## Abstract

**Highlights:**

1. This *VdPRMT1* is a conserved arginine methyltransferase in *Verticillium dahliae*.
2. *VdPRMT1* contributes to fungal growth, stress adaptation, carbon utilization, and virulence.
3. HIGS-mediated silencing of *VdPRMT1* reduces Verticillium wilt severity in cotton.
4. VdPRMT1 interacts with VdLuc7, indicating a potential link to RNA processing.

**Abstract:**

Protein arginine methyltransferases (PRMTs) are key regulators of diverse cellular processes in eukaryotes, including transcriptional regulation, RNA processing, signal transduction and DNA repair. However, the biological functions of PRMTs in *Verticillium dahliae* remain largely unexplored. In this study, we identified a PRMT1 homolog in *V. dahliae*. Targeted deletion of *VdPRMT1* resulted in severely impaired hyphal growth, sporulation, stress responses and pathogenicity. Subcellular localization analysis showed that VdPRMT1 is distributed in both the nucleus and cytoplasm of hyphae. Host-induced gene silencing (HIGS) of *VdPRMT1* in cotton significantly reduced disease severity, supporting its important role in pathogenicity. Furthermore, VdLuc7, a U1 snRNP-associated protein containing multiple RG/RGG motifs, was identified as a putative interacting partner of VdPRMT1 through yeast two-hybrid (Y2H) screening, bimolecular fluorescence complementation (BiFC) and luciferase complementation imaging (LCI) assays. Together, our results demonstrate that *VdPRMT1* is required for normal fungal development and full virulence in *V. dahliae*, and suggest that arginine methylation may contribute to pathogenicity through regulation of RNA processing-related pathways. These findings provide new insights into the molecular mechanisms underlying fungal virulence and identify *VdPRMT1* as a potential target for disease control.

## 1. Introduction

*Verticillium dahliae*, a soil-borne phytopathogenic fungus with strong pathogenicity, causes severe vascular disease in plants [1]. It possesses an exceptionally broad host range, infecting over 600 species including annual/perennial herbs and woody plants [2]. Many economically important crops—such as cotton, tomatoes, potatoes, chili peppers, and sunflowers—are threatened by this pathogen, resulting in substantial global yield losses [3]. Due to its ability to colonize the host vascular system and persist in soil as durable microsclerotia, conventional fungicides are largely ineffective against *V. dahliae* [4]. However, understanding the molecular mechanisms underlying its pathogenicity remains essential for developing effective disease control strategies.

Protein arginine methyltransferases (PRMTs) are evolutionarily conserved enzymes that catalyze the transfer of methyl groups from S-adenosyl-L-methionine (SAM) to arginine residues, generating methylated arginine and S-adenosyl-L-homocysteine (SAH) [5]. This post-translational modification plays critical roles in a wide range of cellular processes, including chromatin remodeling, gene transcription, DNA repair, apoptosis, and signal transduction [6,7,8]. Based on their catalytic activities, PRMTs are generally classified into three major types, including type I enzymes that produce asymmetric dimethylarginine, type II enzymes that generate symmetric dimethylarginine, and type III enzymes that catalyze monomethylarginine formation [9,10,11]. Among them, PRMT1 is widely recognized as the predominant type I PRMT in many eukaryotes and serves as a major source of cellular asymmetric arginine dimethylation [12].

Increasing evidence has demonstrated that PRMTs play important roles in fungal growth, development, and pathogenicity [13,14,15]. In several plant pathogenic fungi, PRMT1 homologs are required for vegetative growth, sporulation, stress adaptation, and virulence. For example, in *Magnaporthe oryzae*, the PRMT1 homolog MoHMT1 is required for normal fungal development and infection, and is involved in pre-mRNA splicing and autophagy-related processes [16]. Similarly, in *Fusarium graminearum*, deletion of the PRMT1 homolog AMT1 leads to defects in hyphal growth, stress responses, and pathogenicity [17]. In *Aspergillus* species, PRMT1 homologs have been implicated in fungal development, oxidative stress responses, catabolic regulation, detoxification processes, and secondary metabolism [18,19,20]. In addition, PRMT-mediated regulation has also been reported in oomycete pathogens such as *Phytophthora capsici*, further suggesting that arginine methylation represents a conserved regulatory mechanism associated with pathogenicity [21]. However, the roles of PRMTs in *V. dahliae* remain largely unknown. In particular, whether PRMT1 contributes to fungal development, stress adaptation, and virulence, and through which molecular mechanisms it functions, has not been systematically investigated.

In this study, we identified four PRMT family members in *V. dahliae* and focused on the functional characterization of VdPRMT1, a homolog of yeast HMT1. Through genetic, phenotypic, and transcriptomic analyses, we demonstrate that *VdPRMT1* is required for fungal growth, stress responses, carbon utilization, and pathogenicity. Furthermore, we show that VdPRMT1 interacts with the spliceosome-associated protein VdLuc7, suggesting a potential link between arginine methylation and RNA processing in *V. dahliae*. These findings provide new insights into the regulatory roles of PRMT1 in fungal biology and pathogenicity.

## 2. Materials and Methods

### 2.1. Growth Conditions of Fungal Strains and Plants

The defoliating *V. dahliae* isolate V991 was used throughout this study. Fungal cultures were maintained at 28 °C for 7–10 days on potato dextrose agar (PDA; 200 g/L potato, 20 g/L dextrose, and 20 g/L agar) or in complete medium (CM; 6 g/L yeast extract, 6 g/L casein acid hydrolysate, and 10 g/L sucrose; pH 7.2), as required for each experiment. Seeds of *Gossypium hirsutum* L. cv. Coker 312 (No. CN-ZM-06186, https://www.cottongen.org/bio_data/18869, accessed on 27 July 2026), were germinated, transferred to a sterilized 1:1 mixture of nutrient soil and vermiculite, and grown at 25 °C under a 16 h-light/8 h-dark regime. Seedlings bearing two cotyledons were used in subsequent assays. *Agrobacterium tumefaciens* strains EHA105 and GV3101 were propagated at 28 °C with shaking at 200 rpm in Luria–Bertani medium (10 g/L tryptone, 5 g/L yeast extract, and 10 g/L NaCl) supplemented with kanamycin sulfate and rifampicin. Cells were collected for transformation when the cultures reached an OD_600_ of 0.5–0.6.

### 2.2. Bioinformatics Analysis

Candidate PRMT proteins in *V. dahliae* were identified by querying the VdLs. 17 genomes in Ensembl Fungi with PRMT amino acid sequences from *Saccharomyces cerevisiae*. Homologous sequences from *V. dahliae*, *Homo sapiens*, *S. cerevisiae*, *Fusarium graminearum*, *Neurospora crassa*, and *Aspergillus nidulans* were aligned with ClustalW. A neighbor-joining phylogeny was then inferred in MEGA 5.2.2. The three-dimensional structure of VdPRMT1 was modeled with ColabFold using the AlphaFold2/MMseqs2 workflow (https://colab.research.google.com/github/sokrypton/ColabFold/blob/main/AlphaFold2.ipynb, accessed on 27 July 2026).

### 2.3. Isolation of DNA and RNA, PCR, RT-qPCR

Total RNA was purified with the RNAprep Pure Polysaccharide Polyphenol Plant Total RNA Extraction Kit (Tiangen, Beijing, China), and first-strand cDNA was generated with HiScript III RT SuperMix for qPCR (+gDNA wiper; Vazyme, Nanjing, China). Genomic DNA was prepared using the Plant Genomic DNA Extraction Kit (Tiangen). DNA and RNA concentrations were measured with a NanoDrop 2000 spectrophotometer (Thermo Fisher Scientific, Waltham, MA, USA). All procedures were performed according to the respective manufacturers’ instructions.

Conventional PCR mixtures contained 2× Phanta Flash Master Mix (Vazyme, Nanjing, China). Amplification comprised 3 min at 95 °C; 35 cycles of 30 s at 95 °C, 30 s at 58 °C, and 1 min at 72 °C; and a final 5 min extension at 72 °C. Products were resolved on 1% agarose gels. Each reaction was performed in three technical replicates, and all primers are listed in Table S1.

For transcript quantification, reactions were prepared with ChamQ Universal SYBR qPCR Master Mix (Vazyme, Nanjing, China) following the supplier’s recommendations. Three independent biological samples were analyzed for each treatment. Expression values were normalized to *V. dahliae* actin (*VDAG_00941*) and calculated by the 2^−ΔΔCt^ method. Primer information is provided in Table S1.

### 2.4. Mutant Construction

To generate the *VdPRMT1* deletion construct, approximately 1 kb genomic regions immediately upstream and downstream of *VdPRMT1* were PCR-amplified from V991. The two flanking products were assembled with linearized pCHG using the ClonExpress Ultra One Step Cloning Kit (Vazyme, Nanjing, China), yielding the knockout plasmid illustrated in Figure S1A. Transformants were subjected to three rounds of 5-FU-based negative selection before PCR confirmation.

For complementation, the *VdPRMT1* coding sequence was inserted into pMC by seamless cloning (Figure S1B). The deletion and complementation plasmids were separately introduced into *A. tumefaciens* EHA105. *VdPRMT1* knockout and complemented strains were subsequently obtained through *A. tumefaciens*-mediated transformation.

### 2.5. Subcellular Localization

The pMC-VdPRMT1 complementation plasmid was transferred into the *VdPRMT1* deletion background by *A. tumefaciens*-mediated transformation. G418-resistant colonies were examined by PCR with EGFP-specific and *VdPRMT1* internal primer pairs. Two isolates positive for both targets (C1-5 and C1-6) were retained for localization analysis (Figure S1B). The mycelia of the VdPRMT1-complemented strain were thawed, and 500 µL of the resulting spore suspension was added to 50 mL CM. Following 3 days of incubation at 25 °C and 200 rpm, mycelia were stained with 5 μg/mL DAPI solution (Coolaber, Beijing, China) for 10 min. The samples were then rinsed twice in PBS solution before observation. Then fluorescence was visualized with a Zeiss LSM 980 laser-scanning confocal microscope.

### 2.6. Observation of Fungal Phenotype

Spore suspensions were prepared from V991, the *VdPRMT1* deletion mutants, and the complemented strains. For each assay, a 10 µL droplet was placed on PDA, defined media containing individual carbon sources, or PDA supplemented with the indicated stress compound. Plates were incubated in darkness at 25 °C for 12 days. To determine conidial production, each colony was flooded with 1 mL sterile water and gently scraped; the recovered suspension was passed through a 40 µm cell strainer, and conidia in the filtrate were counted with a hemocytometer.

### 2.7. Fungal Inoculation and Pathogenicity Determination

Cotton pathogenicity was evaluated with spore suspensions of V991, the *VdPRMT1* knockout mutants, and the complemented strains. When cotton seedlings developed two true leaves, root dip inoculation was performed; seedlings were gently uprooted to minimize root damage, immersed in the respective spore suspensions for 5 min, and replanted into sterile soil. Disease incidence was evaluated 30 d post-inoculation; the disease index was calculated as [Σ (diseased plants at each grade × grade)/(10 × 4)] × 100 and photographed. Subsequently, a 2 cm stem segment below the cotyledonary node was longitudinally sectioned; vascular discoloration was observed and imaged under a stereomicroscope. Statistical analysis was performed using one-way ANOVA followed by Tukey’s multiple comparison test. Significance was indicated using asterisks (* *p* < 0.05, ** *p* < 0.01, *** *p* < 0.001).

### 2.8. RNA Sequencing (RNA-Seq) Analysis

To simulate osmotic stress conditions, wild-type and Δ*VdPRMT1* strains were grown in potato dextrose broth (PDB) medium supplemented with sorbitol under shaking conditions (150 rpm) at 25 °C. Mycelia were collected after incubation for RNA extraction and transcriptome sequencing. Three independent biological replicates were included for each strain. Total RNA was extracted using a standard fungal RNA extraction protocol, and RNA quality was assessed prior to library construction. RNA-seq libraries were constructed and sequenced on an Illumina high-throughput sequencing platform.

Raw reads were subjected to quality control using standard filtering procedures to remove adapter sequences and low-quality reads. Clean reads were aligned to the *V. dahliae* reference genome using HISAT2 v2.2.1. Gene expression levels were quantified and normalized, and differential expression analysis between WT and Δ*VdPRMT1* strains was performed using DESeq2 v1.36.0.

Genes with DESeq2 adjusted *p* values (padj) ≤ 0.05 and an absolute log2 fold change (|log2FoldChange|) ≥ 1.0 were considered differentially expressed genes (DEGs). Functional enrichment analyses, including Gene Ontology (GO) and Kyoto Encyclopedia of Genes and Genomes (KEGG) analyses, were performed based on the identified DEGs to determine significantly enriched biological processes and pathways.

### 2.9. Stress and Carbon Utilization Assays

Vegetative growth of wild-type V991, Δ*VdPRMT1*, and complemented strains was evaluated under different stress and carbon source conditions. For stress tolerance assays, PDA medium was supplemented with different stress-inducing agents, including Congo red (CR, 75 μg/mL), calcofluor white (CFW, 30 μg/mL), sodium dodecyl sulfate (SDS, 0.01%, *w*/*v*), sorbitol (SBT, 182.17 mg/mL), and sodium chloride (NaCl, 40.95 mg/mL). All additives were prepared as sterile stock solutions and added to autoclaved PDA medium after cooling to approximately 55 °C prior to plate pouring. For carbon utilization assays, strains were first grown in complete medium (CM), and conidia were collected by filtration through a sterile 40 μm cell strainer. A 10 μL aliquot of conidial suspension (2 × 10^6^ spores/mL) was spotted onto Czapek Dox agar plates supplemented with different sole carbon sources, including sucrose (30 g/L), pectin (10 g/L), xylose (10 g/L), starch (17 g/L), and galactose (10 g/L). PDA plates without additional carbon sources were used as controls.

All plates were incubated at 25 °C in the dark. Colony morphology was photographed after 14 days of incubation, and colony diameters were measured. Each experiment included five biological replicates per condition. Growth inhibition was calculated by comparing colony diameters of mutant strains with those of the wild-type strain.

### 2.10. Host-Induced Gene Silencing (HIGS)

According to the VdLs. 17 cDNA database, a 400–500 bp *VdPRMT1* CDS fragment was PCR-amplified (Primer 5) and ligated into pTRV2. The resulting pTRV2-VdPRMT1 and empty vectors were transformed into *Agrobacterium* GV3101. Cotyledons were syringe-infiltrated with the resuspended cells, dark-incubated, then returned to photoperiod. Thirty days post-inoculation (dpi), disease index, *VdPRMT1* silencing efficiency and fungal biomass were assessed to evaluate the gene’s role in pathogenesis [22,23,24].

### 2.11. Yeast Two-Hybrid

*VdPRMT1* and *VdLuc7* coding regions were inserted into pGBKT7 (Figure S1C) and pGADT7 (Figure S1D), respectively. The bait and prey plasmids were first transformed individually into *Saccharomyces cerevisiae* AH109, and transformants were recovered on SD/−Trp or SD/−Leu. BD-VdPRMT1 and AD-VdLuc7 were then introduced together. Co-transformants were incubated at 30 °C for 3–5 days on SD/−Trp/−Leu and SD/−Trp/−Leu/−His/−Ade. An interaction was considered specific when growth occurred on the quadruple-dropout medium for the test pair but not for the negative controls.

### 2.12. Bimolecular Fluorescence Complementation

For bimolecular fluorescence complementation, the *VdPRMT1* coding sequence and a stop-codon-free VdLuc7 coding sequence were cloned into pB-NEO-VN and pB-HPT-VC, respectively (Figure S1E,F). *V. dahliae* V991 protoplasts received either pB-NEO-VN-VdPRMT1 together with pB-HPT-VC-VdLuc7 or the corresponding empty-vector control pair. Transformants were recovered on PDA containing G418 and hygromycin B and purified through repeated dual-antibiotic selection. Reconstituted YFP fluorescence was then examined microscopically.

### 2.13. Luciferase Complementation Imaging Assay

The coding sequences of *VdPRMT1* and *VdLuc7* were cloned into the corresponding nLUC and cLUC vectors to generate VdLuc7-nLUC and VdPRMT1-cLUC constructs. The recombinant plasmids, as well as the empty vector controls, were introduced into *A. tumefaciens* strain GV3101. *Agrobacterium* cultures carrying the indicated constructs were grown overnight, collected by centrifugation, and resuspended in infiltration buffer to an appropriate optical density. Equal volumes of *Agrobacterium* suspensions carrying the indicated plasmid combinations were mixed at a 1:1 ratio and co-infiltrated into the leaves of 4-week-old *Nicotiana benthamiana* plants. After infiltration, the plants were kept in the dark for 24 h and then transferred to normal growth conditions for another 24 h. At 48 h post-infiltration, the infiltrated leaves were sprayed with 1 mM luciferin and kept in the dark for several minutes before luminescence detection. LUC signals were captured using a Nightshade LB985 imaging system (Berthold Technologies, Bad Wildbad, Germany).

### 2.14. Statistical Analysis

All quantitative analyses were conducted in GraphPad Prism 9.4.1.458. Colony diameter and conidial yield were determined from three independent biological experiments and are reported as the mean ± standard deviation. Differences among more than two groups were tested by one-way ANOVA, followed by Tukey’s multiple-comparison procedure when the overall test was significant.

## 3. Results

### 3.1. Phylogeny, Structure and Expression Characteristics of VdPRMT1

**Figure 1:**
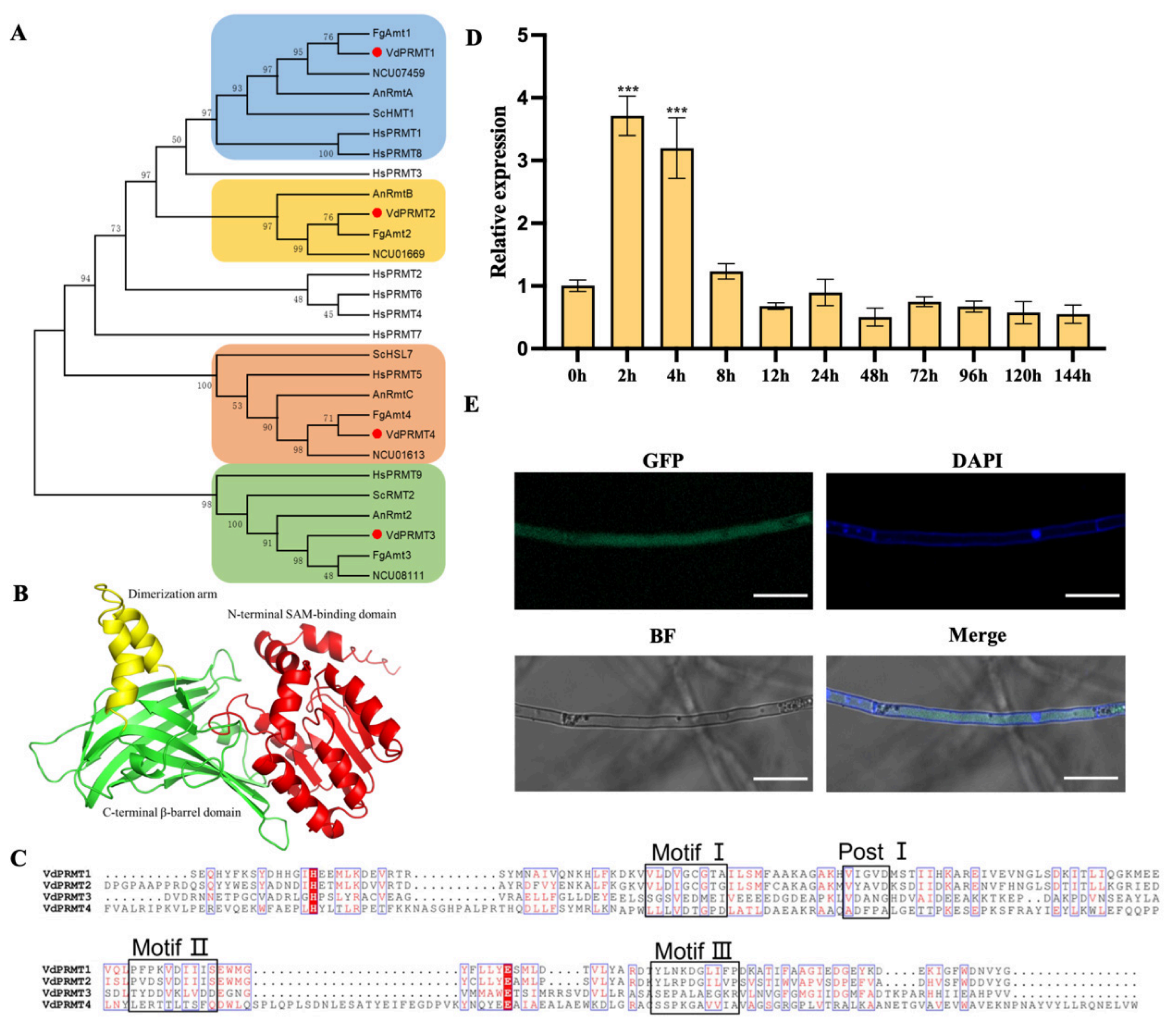
Phylogenetic, structure, conserved domain, subcellular localization and expression pattern analysis of VdPRMT1 in *V. dahliae*. (**A**) Phylogenetic tree of the PRMT family illustrating the evolutionary relationship among VdPRMT1 and orthologs from other species. Red circles indicate the four VdPRMT proteins identified in *V. dahliae*. (**B**) Predicted tertiary structure of VdPRMT1, with labeled dimerization arm, N-terminal SAM-binding domain, and C-terminal β-barrel domain. The predicted model showed high confidence, with a pTM score of 0.91 and high pLDDT values for most residues within the structured regions, supporting the reliability of the predicted global fold and local structural features. (**C**) Multiple sequence alignment of PRMT pr oteins across species, highlighting conserved motifs. Boxed residues indicate conserved amino acids, and Motif I, Post I, Motif II, and Motif III are indicated above the corresponding regions. (**D**) Relative expression of *VdPRMT1* at different hours post-inoculation (hpi) during cotton infection, as determined by RT-qPCR. Error bars represent standard deviation based on three independent replicates. (*** *p* < 0.001, one-way ANOVA). (**E**) Subcellular localization of VdPRMT1 in hyphae of *V. dahliae*. Scale bar: 10 µm.

Four protein arginine methyltransferases (PRMTs) were identified in *V. dahliae*, designated here as *VdPRMT1* (VDAG_08751), *VdPRMT2* (VDAG_04075), *VdPRMT3* (VDAG_04088) and *VdPRMT4* (VDAG_02006). To elucidate the evolutionary relationship between VdPRMTs and other fungal PRMTs, a phylogenetic analysis was conducted. The results indicated that VdPRMT1 clustered within the same clade as PRMTs from *N. crassa* and *F. graminearum*, suggesting a conserved evolutionary position within the eukaryotic PRMT family (Figure 1A). AlphaFold-based structural prediction (Figure 1B) revealed that VdPRMT1 adopts a typical PRMT architecture, consisting of a dimerization arm, an N-terminal Rossmann-like SAM-binding domain, and a C-terminal β-barrel domain. The predicted model showed high structural confidence (pTM score = 0.91). Sequence alignment of VdPRMTs (Figure 1C) further identified a conserved methyltransferase domain encompassing Motif I, Post I, Motif II and Motif III.

Gene expression dynamics analyzed by RT-qPCR (Figure 1D) revealed significant upregulation of *VdPRMT1* during early infection stages, suggesting a potential role in the early phase of *V. dahliae* infection in cotton. Subcellular localization results (Figure 1E) demonstrated that the VdPRMT1-GFP fusion protein was uniformly distributed in both the nucleus and cytoplasm of hyphae, while forming granular aggregates in conidia, implying a potential role in conidium-specific physiological processes.

### 3.2. VdPRMT1 Positively Regulates the Pathogenicity of V. dahliae

**Figure 2:**
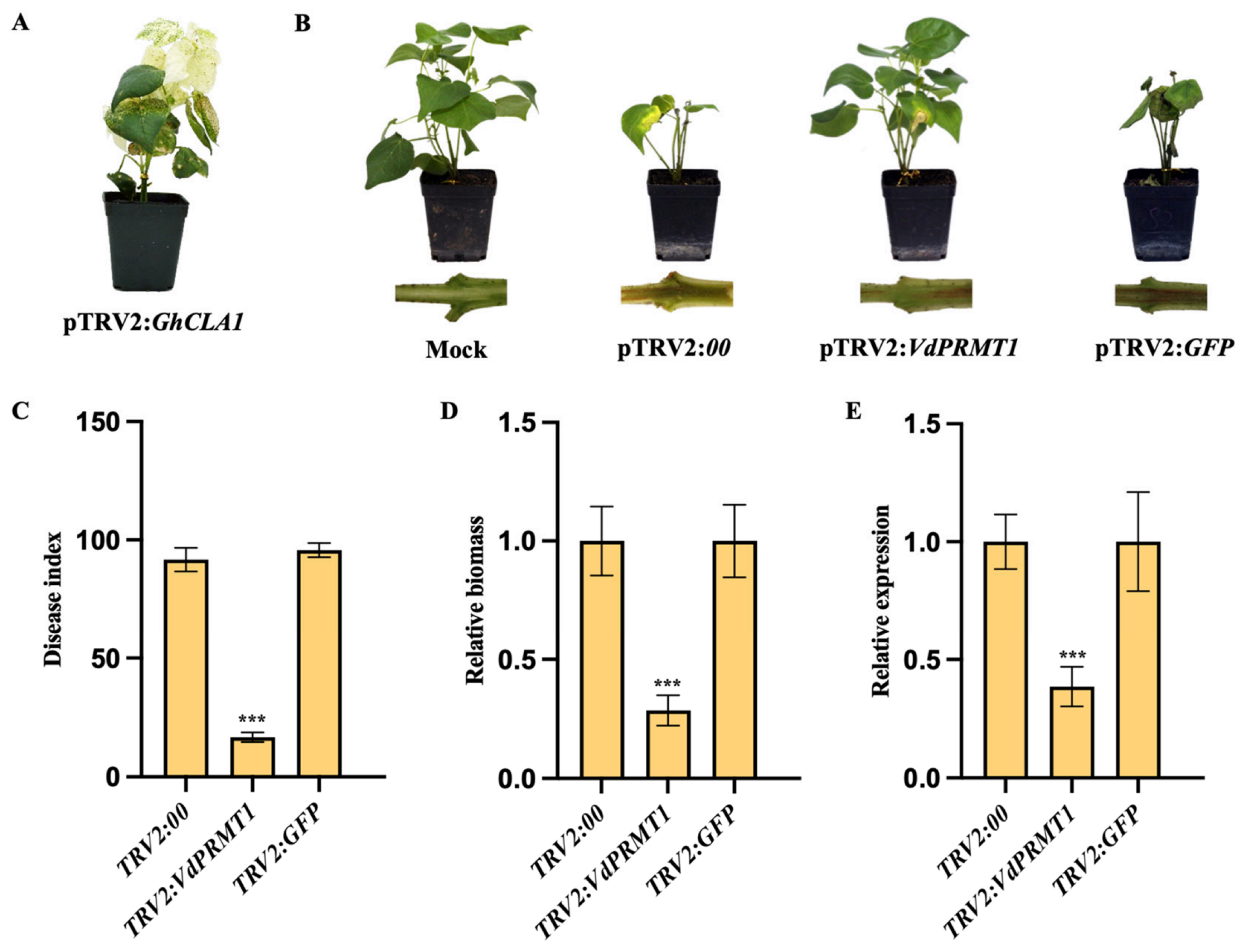
Pathogenicity of *V. dahliae* following HIGS-mediated transient silencing of *VdPRMT1*. (**A**) Albino phenotype in cotton plants infiltrated with *Agrobacterium* carrying pTRV2-*GhCLA1*. (**B**) Disease symptoms in cotton after *VdPRMT1* silencing and subsequent inoculation with wild-type *V. dahliae* strain V991 at 30 days. (**C**) Disease index of cotton at 30 dpi. (**D**) Fungal biomass in cotton tissues at 30 dpi. (**E**) Expression level of *VdPRMT1* in cotton at 30 dpi. Error bars represent standard deviation based on three independent replicates. (***, *p* < 0.001, one-way ANOVA).

Transient silencing of *VdPRMT1* was achieved in cotton HIGS. Cotton plants infiltrated with *Agrobacterium* carrying the pTRV2-*GhCLA1* plasmid exhibited an albino phenotype (Figure 2A). Compared to controls infiltrated with empty pTRV2 and pTRV2-GFP, plants treated with pTRV2-*VdPRMT1* showed significantly alleviated symptoms (Figure 2B), accompanied by an approximately 81.82% reduction in disease index (Figure 2C). Concurrently, the expression level of *VdPRMT1* (Figure 2E) and fungal biomass (Figure 2D) decreased significantly by 61.43% and 71.43%, respectively. These results demonstrate that *VdPRMT1* was successfully silenced by HIGS and substantiate its critical role in the pathogenicity of *V. dahliae* during cotton infection.

### 3.3. VdPRMT1 Affects the Physiological Phenotype of V. dahliae by Regulating Growth, Sporulation

**Figure 3:**
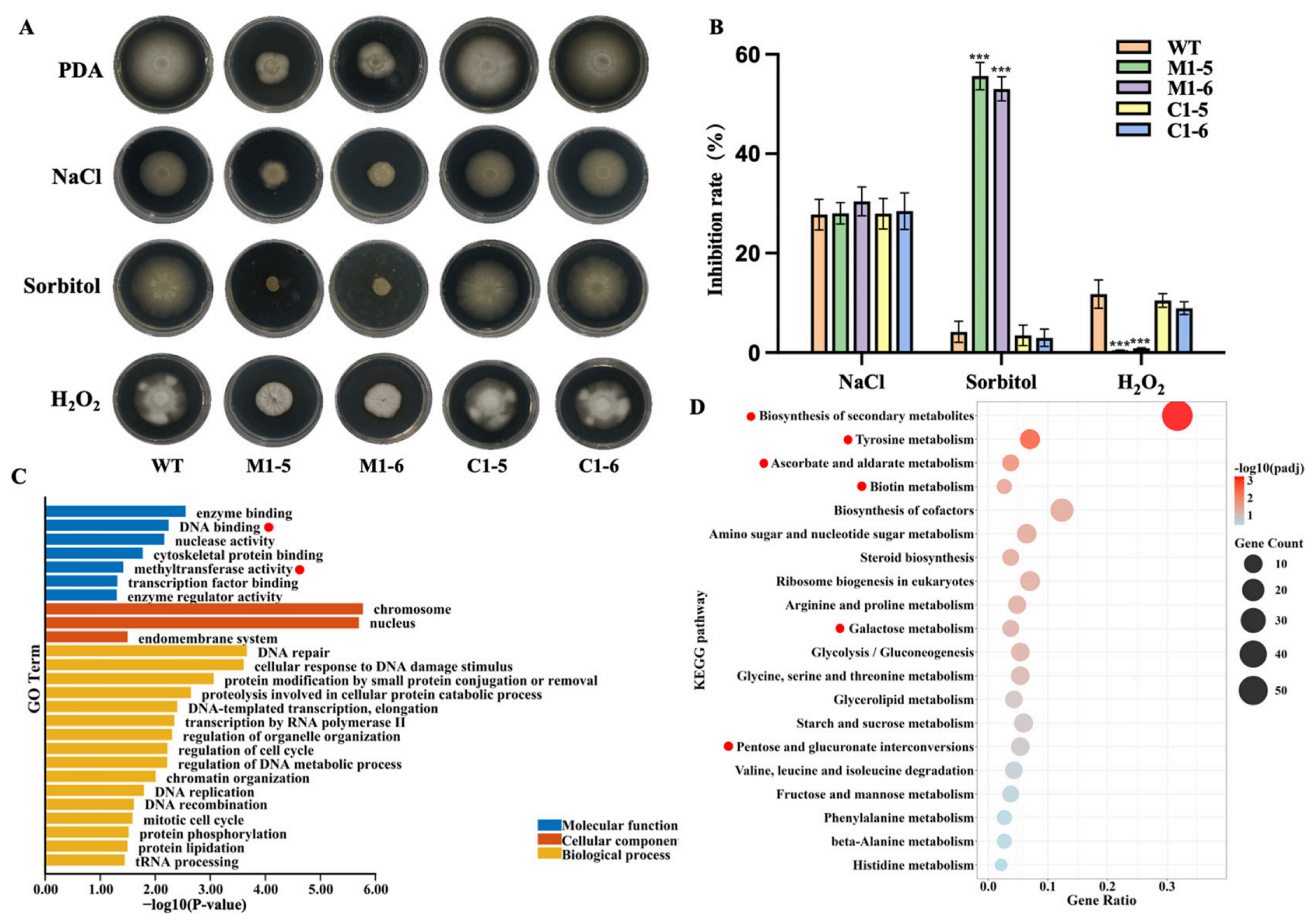
Phenotypic and transcriptome analysis of *VdPRMT1* knockout mutant strains in *V. dahliae*. (**A**) Colony phenotypes of the indicated strains grown on PDA or PDA containing NaCl, Sorbitol and H_2_O_2_ for 12 days at 25 °C. (**B**) Bar chart showing colony diameters in (A) were measured. Error bars represent standard deviation based on three independent replicates. (***, *p* < 0.001; one-way ANOVA). (**C**) GO enrichment analysis of DEGs identified in the Δ*VdPRMT1* mutant relative to the wild-type strain. The significantly enriched GO terms were mainly related to enzyme binding, DNA binding, chromosome, nucleus, DNA repair, and transcription. (**D**) KEGG pathway enrichment analysis of DEGs identified in the Δ*VdPRMT1* mutant relative to the wild-type strain.

Following transient silencing of *VdPRMT1*, which indicated its involvement in the pathogenicity of *V. dahliae*, we further investigated its biological function by generating *VdPRMT1* knockout mutants (M1-5, M1-6) and functional complementation strains (C1-5, C1-6) (Figures S2 and S3). Phenotypic comparisons were conducted among wild-type (WT), mutants, and complemented strains. On PDA medium, the mutants exhibited significantly reduced colony diameter (Figure 3A) and sporulation (Figure S4A) compared to WT, while the complemented strains largely restored the wild-type phenotype, indicating that *VdPRMT1* regulates hyphal growth and conidiation. Under stress conditions induced by NaCl, Sorbitol and H_2_O_2_, the mutants showed altered inhibition rates of colony growth (Figure 3A,B) and spore production (Figure S4A) relative to WT, demonstrating involvement of *VdPRMT1* in osmotic and oxidative stress responses.

To investigate the molecular mechanisms underlying VdPRMT1-regulated growth in *V. dahliae*, we conducted transcriptome sequencing of the *VdPRMT1* knockout mutant (PRMT1_SBT) and the wild-type strain (WT_SBT) cultured in sorbitol-supplemented medium. As shown in the volcano plot, 793 genes were significantly up-regulated and 711 genes were significantly down-regulated (Figure S4B). GO enrichment analysis further categorized the DEGs, with notable enrichment in molecular functions such as enzyme binding, DNA binding, and methyltransferase activity (Figure 3C), suggesting that *VdPRMT1* may be associated with enzyme complex assembly and epigenetic regulation. KEGG pathway enrichment analysis revealed that deletion of *VdPRMT1* led to significant alterations in multiple metabolic and cellular pathways. Notably, pathways related to carbohydrate metabolism and secondary metabolite biosynthesis, including galactose metabolism, pentose and glucuronate interconversions, ascorbate and aldarate metabolism, tyrosine metabolism, and biotin metabolism, were significantly enriched. In addition, pathways associated with fundamental cellular processes, such as ribosome biogenesis, amino sugar and nucleotide sugar metabolism, and steroid biosynthesis, were also affected. These results indicate that VdPRMT1 broadly regulates gene expression associated with metabolic processes and genetic information processing in *V. dahliae*, thereby influencing fungal growth, development, and environmental adaptation.

### 3.4. VdPRMT1 Is Required for Cell Wall Integrity and Stress Responses in V. dahliae

**Figure 4:**
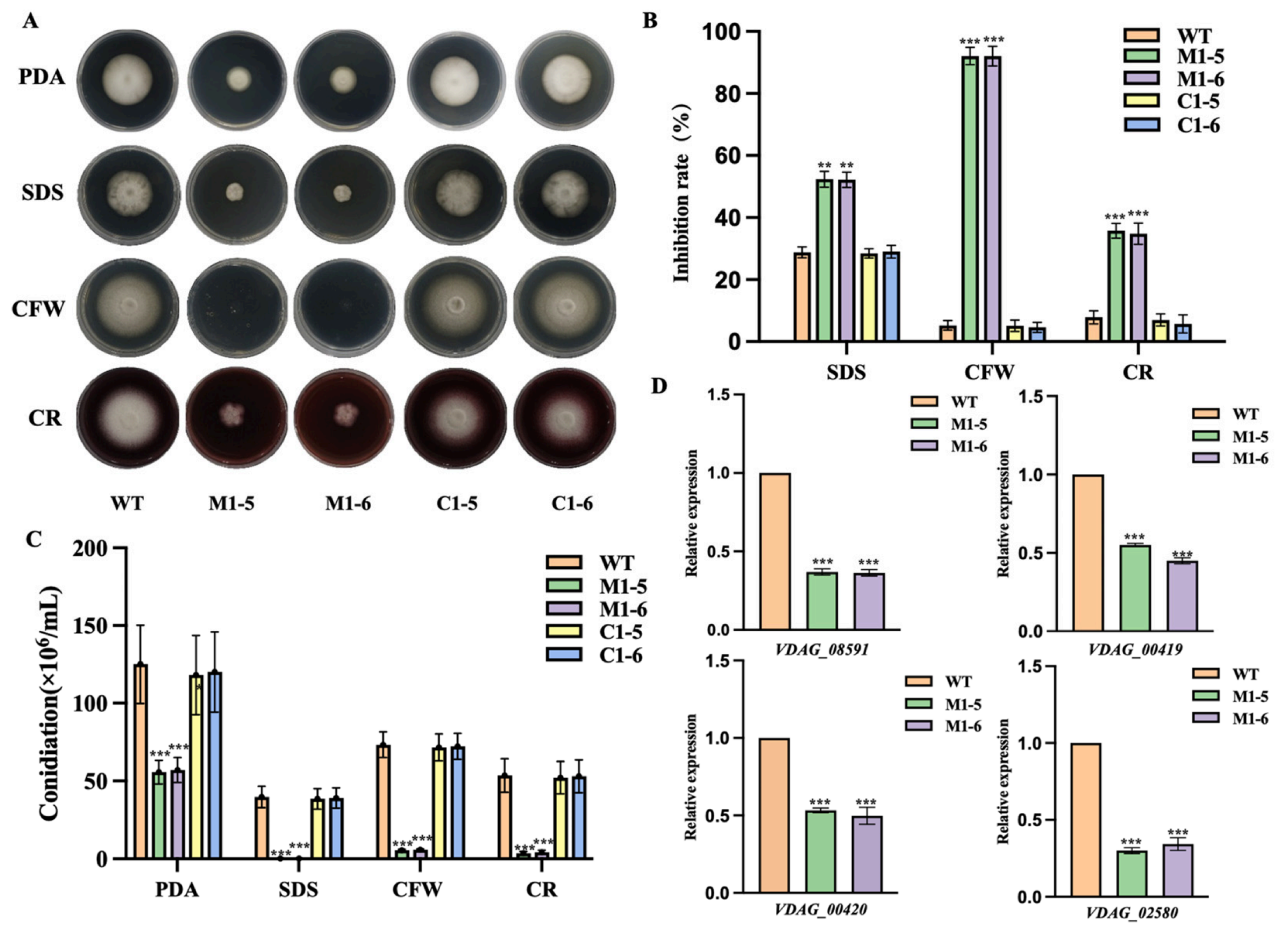
Phenotypic analysis of *VdPRMT1* knockout mutants and complementation strain in *V. dahliae*. (**A**) Colony morphology of WT, mutants, and complementation strains under different cell wall stress conditions after 12 days. (**B**) Colony growth inhibition rate under stress conditions. (**C**) Sporulation quantity under stress medium. (**D**) Relative expression level of four genes (*VDAG_08591, VDAG_00419*, *VDAG_00420*, *VDAG_02580*) related to cell wall biosynthesis in *V. dahliae*. Error bars represent standard deviations of three replicates, and asterisks represent significant differences (*** *p* < 0.001; ** *p* < 0.01). There were three biological experiments performed for each strain and three technical replicates per biological experiment.

Under cell wall stress conditions induced by SDS, CFW and CR, the mutants showed altered inhibition rates of colony growth (Figure 4A,B) and spore production (Figure 4C) relative to WT, demonstrating involvement of *VdPRMT1* in cell wall stress responses. To further elucidate the molecular basis underlying the increased stress sensitivity, the expression levels of several genes involved in cell wall biosynthesis were analyzed. RT-qPCR results showed that multiple chitin synthase–encoding genes, including *VDAG_08591*, *VDAG_02580*, *VDAG_00419*, and *VDAG_00420*, were significantly downregulated in the Δ*VdPRMT1* mutant compared with the wild type (Figure 4D). These results suggest that VdPRMT1 is required for maintaining cell wall integrity, likely through transcriptional regulation of chitin biosynthesis–related genes, thereby contributing to fungal tolerance to cell wall–perturbing stresses.

### 3.5. VdPRMT1 Regulates Carbon Utilization in V. dahliae

**Figure 5:**
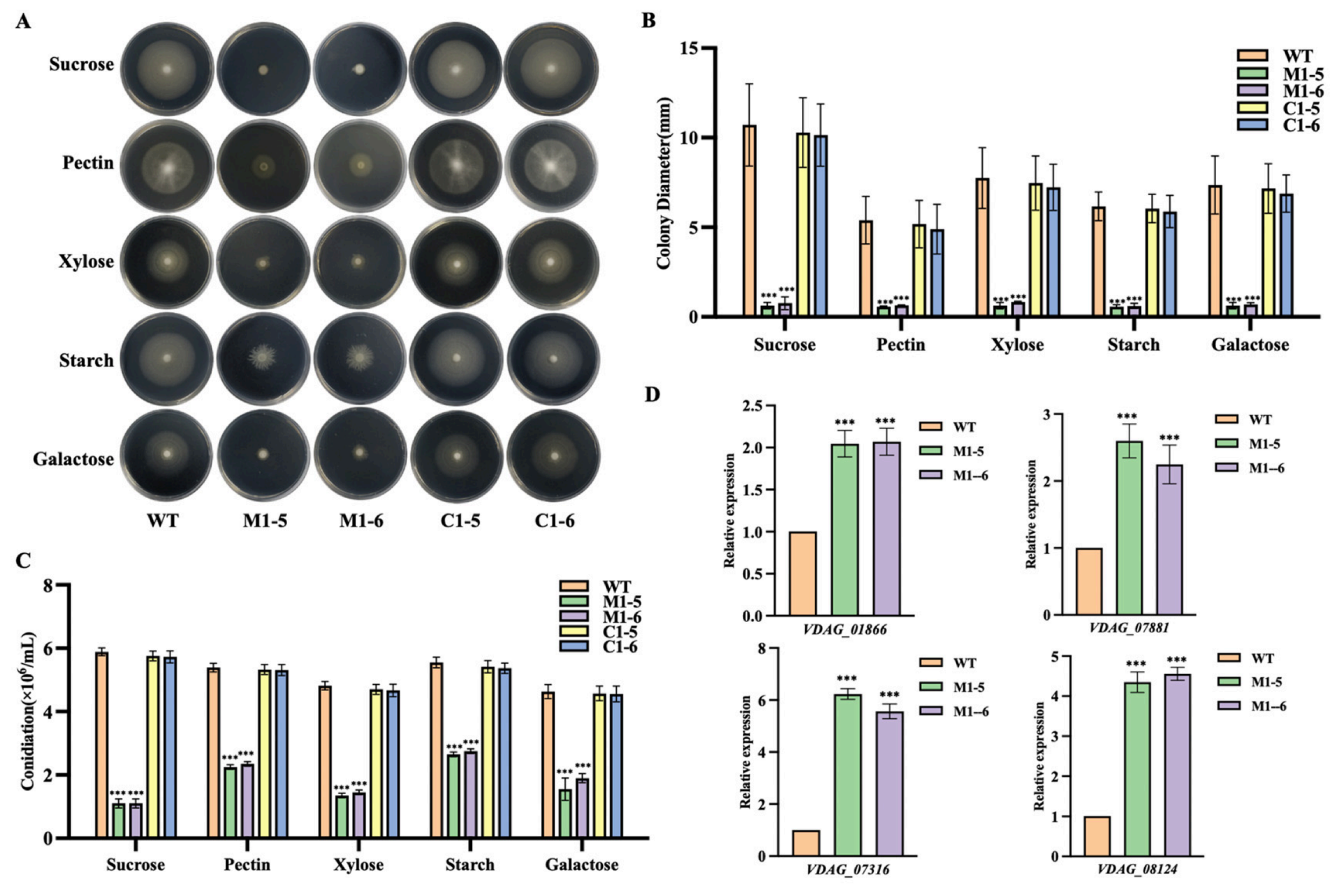
VdPRMT1 is required for carbon utilization in *V. dahliae*. (**A**) Colony morphology of WT, mutants, and complementation strains grown on different carbon sources for 12 days. (**B**) Colony diameter on different carbon sources. (**C**) Sporulation quantity on different carbon sources. (**D**) Relative expression level of four genes (*VDAG_01866, VDAG_07881*, *VDAG_07316*, *VDAG_08124*) related to carbohydrate metabolism in *V. dahliae*. Error bars represent standard deviations of three replicates, and asterisks represent significant differences (***, *p* < 0.001). There were three biological experiments performed for each strain and three technical replicates per biological experiment.

KEGG enrichment analysis revealed that multiple carbohydrate metabolism pathways were significantly altered in the Δ*VdPRMT1* mutant, suggesting a role of VdPRMT1 in regulating carbon metabolism. To validate whether these transcriptional changes affected fungal carbon utilization, we assessed the growth of the Δ*VdPRMT1* mutant on media containing different carbon sources. The Δ*VdPRMT1* mutant exhibited significantly impaired colony expansion across all tested carbon sources, indicating a broad defect in carbon utilization (Figure 5A,B). Consistently, sporulation of the mutant was also markedly reduced under these conditions (Figure 5C). In contrast, the complementation strains restored these defects to wild-type levels. These results demonstrate that VdPRMT1 is required for efficient utilization of diverse carbon sources, particularly those derived from plant cell wall components.

To further investigate the molecular basis underlying the impaired carbon utilization, the expression of representative genes involved in carbohydrate metabolism and plant cell wall degradation was analyzed by RT-qPCR. The results showed that genes associated with carbon uptake and polysaccharide degradation, including a sugar transporter (*VDAG_08124*), a pectinesterase (*VDAG_07881*), and an alpha-xylosidase (*VDAG_01866*), were significantly upregulated in the Δ*VdPRMT1* mutant. In addition, a homogentisate 1,2-dioxygenase (*VDAG_07316*) exhibited strong induction (Figure 5D). These results indicate that deletion of *VdPRMT1* leads to dysregulated carbon metabolism, likely accompanied by compensatory activation of carbon acquisition and degradation pathways despite reduced overall growth and carbon utilization efficiency.

### 3.6. VdPRMT1 Is Required for Full Pathogenicity in Cotton

**Figure 6:**
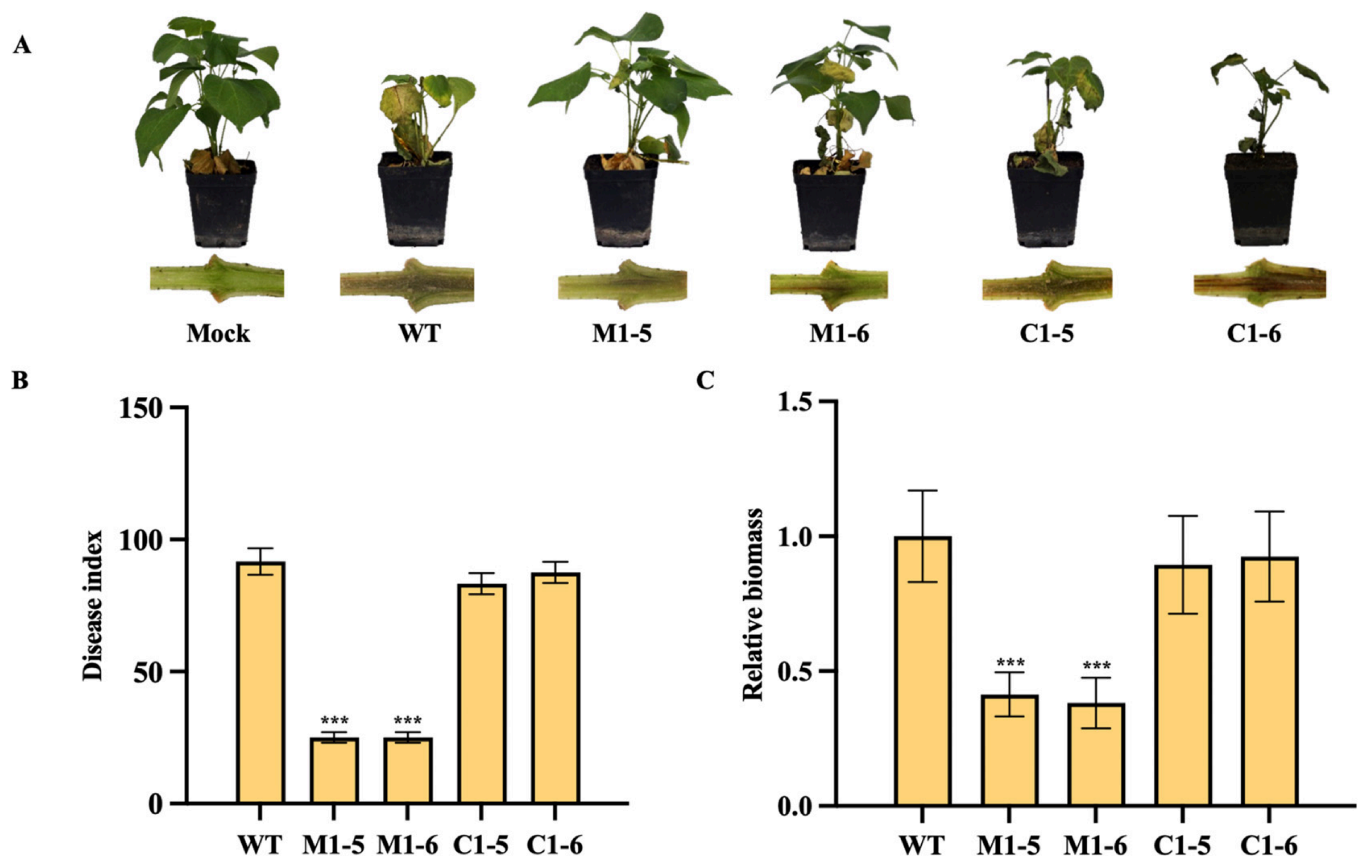
Pathogenicity of *VdPRMT1* deletion mutants. (**A**) Disease symptoms in cotton inoculated with wild-type (V991), *VdPRMT1* knockout mutants, and functional complementation strains of *V. dahliae* 30 dpi. Mock: cotton plants inoculated with sterile water instead of fungal spores. (**B**) Disease index of cotton infected with the indicated strains. (**C**) Fungal biomass in cotton roots at 30 dpi. Error bars represent standard deviations of three replicates, and asterisks represent significant differences (***, *p* < 0.001).

To investigate the effect of *VdPRMT1* deletion on the pathogenicity of *V. dahliae* in cotton, disease indices and fungal biomass were assessed at 30 dpi. Compared to the wild-type strain V991, the two knockout mutants (M1-5 and M1-6) caused a significant reduction in disease index by 72.73% (Figure 6B), and root fungal biomass decreased by 59% and 62%, respectively (Figure 6C), indicating markedly impaired virulence. In contrast, the functional complementation strains C1-5 and C1-6 restored pathogenic phenotypes to wild-type levels (Figure 6A–C), confirming that *VdPRMT1* plays a critical role in regulating the pathogenicity of *V. dahliae*. These results demonstrate that *VdPRMT1* is essential for normal virulence and provide important insights for further elucidating the pathogenic mechanisms of *V. dahliae*.

### 3.7. Screening and Verification of VdPRMT1 Interacting Proteins

**Figure 7:**
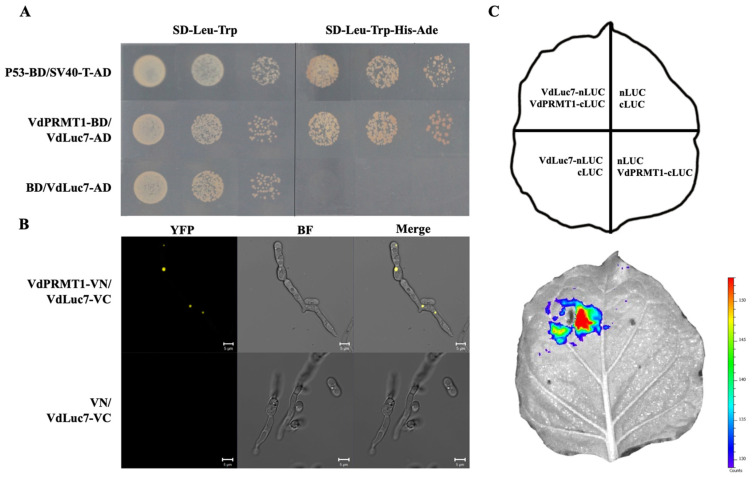
Interaction between VdPRMT1 and VdLuc7 in *V. dahliae*. (**A**) Yeast two-hybrid assay showing growth of yeast co-transformed with pGBKT7-VdPRMT1 and pGADT7-VdLuc7 on SD/–Trp/–Leu/–His/–Ade medium, confirming protein–protein interaction. The positive control (PC) and test combination exhibited growth, while negative pairs did not. (**B**) Bimolecular fluorescence complementation (BiFC) assay detecting YFP signal in *V. dahliae* hyphae co-expressing VN-VdPRMT1 and VC-VdLuc7, indicating in vivo interaction. No fluorescence was observed in the negative control (NC). Scale bars: 5 µm. (**C**) The interaction of VdPRMT1 with VdLuc7 was detected by LCI assay.

To further investigate the molecular basis underlying *VdPRMT1*-mediated pathogenicity, a yeast two-hybrid (Y2H) screen was performed using *VdPRMT1* as bait to identify interacting proteins from a *V. dahliae* V991 cDNA library. A total of 35 candidate proteins interacting with VdPRMT1 were identified. To further evaluate potential PRMT substrates, we analyzed the presence of arginine-glycine-rich (RG/RGG) motifs among the identified candidates, as these motifs are known preferential recognition sites of PRMT family enzymes [25,26,27]. The distribution analysis revealed that most candidate proteins contained varying numbers of RG/RGG motifs, ranging from 0 to 7 (Table S2). Notably, VDAG_05301, annotated as a Luc7 homolog (VdLuc7), contained the highest number of RG/RGG motifs (*n* = 7) among all candidates, suggesting a strong potential for PRMT-dependent interaction. Based on the combination of screening frequency and motif enrichment, VDAG_05301 was selected as a representative candidate for further experimental validation. One-to-one validation (Figure 7A) showed that only yeast co-transformed with pGBKT7-*VdPRMT1* and pGADT7-*VdLuc7* grew on SD/-Trp/-Leu/-His/-Ade medium, confirming a specific interaction between VdPRMT1 and VdLuc7 in yeast.

To further validate this interaction, a bimolecular fluorescence complementation (BiFC) assay was conducted in *V. dahliae*. Strong YFP fluorescence was observed in hyphae co-expressing VdPRMT1-VN and VdLuc7-VC using confocal microscopy (Figure 7B), while no signal was detected in negative controls. To consolidate and validate the interaction identified by Y2H and BiFC, luciferase complementation imaging (LCI) was performed as an orthogonal approach in *Nicotiana benthamiana* leaves. VdPRMT1 and VdLuc7 were fused to the C-terminal (cLUC) and N-terminal (nLUC) fragments of luciferase, respectively, and co-expressed via *Agrobacterium tumefaciens*-mediated transient transformation. As shown in Figure 7C, strong luminescence signals were detected in leaf areas co-expressing VdLuc7-nLUC and VdPRMT1-cLUC, indicating a physical interaction between VdLuc7 and VdPRMT1 in planta. These results provide strong evidence that *VdPRMT1* and *VdLuc7* interact in *V. dahliae*. In addition, repeated attempts to generate *VdLuc7* deletion mutants using both *Agrobacterium tumefaciens*-mediated transformation and PEG-mediated protoplast transformation were unsuccessful (Figure S5), suggesting that VdLuc7 is likely important for fungal viability or growth.

## 4. Discussion

Protein arginine methyltransferases (PRMTs) are evolutionarily conserved enzymes that regulate diverse cellular processes through arginine methylation of histone and non-histone proteins, thereby influencing gene expression, RNA metabolism, and signal transduction [10,28]. In this study, we identified four PRMT family members in *V. dahliae* and characterized the biological function of VdPRMT1, a homolog of *S. cerevisiae* HMT1. Our results demonstrate that VdPRMT1 plays an important role in fungal growth and pathogenicity. Subcellular localization analysis showed that VdPRMT1 is distributed in both the nucleus and cytoplasm of hyphae, suggesting that it may participate in multiple regulatory processes. Notably, VdPRMT1 formed granular structures in spores, which resemble autophagy-related structures reported in *M. oryzae* [16]. Given the essential role of autophagy in cellular homeostasis and stress responses [29,30], this observation raises the possibility that VdPRMT1 may be associated with autophagy-related pathways, although further investigation is required. Deletion of VdPRMT1 resulted in significant defects in hyphal growth and conidiation, indicating that VdPRMT1 is required for normal fungal development. Similar phenotypes have been reported in other filamentous fungi, including *M. oryzae* and *F. graminearum*, where PRMT1 homologs are essential for vegetative growth and reproduction [16,17]. These findings suggest that the role of PRMT1 in regulating fungal development is conserved across different fungal species.

The Δ*VdPRMT1* mutant exhibited increased sensitivity to cell wall stress agents, suggesting that VdPRMT1 plays an important role in maintaining cell wall integrity. Previous studies have shown that PRMT1 homologs are involved in responses to oxidative and osmotic stress in filamentous fungi, such as *Aspergillus flavus*, where arginine methylation is required for maintaining cellular integrity under stress conditions [20,31]. In plant pathogenic fungi, the cell wall is essential for maintaining cellular structure and protecting against host-derived stresses, and defects in cell wall integrity are often associated with reduced virulence [16,32,33,34,35]. In *Verticillium dahliae*, previous studies have also demonstrated that genes involved in chitin synthesis and cell wall integrity are required for normal growth and infection, further highlighting the importance of this pathway in fungal virulence.

Transcriptome analysis revealed that deletion of *VdPRMT1* led to widespread changes in gene expression, particularly in pathways related to metabolism and genetic information processing. These findings are consistent with previous studies showing that PRMT1 influences global gene expression through both transcriptional and post-transcriptional regulation [36]. Disruption of these processes is likely to affect multiple cellular functions, thereby contributing to the observed defects in growth and pathogenicity. Consistent with transcriptomic alterations, carbon source utilization assays showed that the Δ*VdPRMT1* mutant exhibited impaired growth and sporulation when sucrose and pectin were used as carbon sources. These results suggest that VdPRMT1 is required for efficient utilization of plant-derived carbon sources. Similar observations have been reported in other pathogenic fungi, where defects in nutrient acquisition are closely associated with reduced virulence [37,38]. In plant pathogenic fungi, efficient utilization of host-derived carbohydrates, including complex polysaccharides such as pectin and cellulose, is critical for successful colonization. Studies in multiple plant pathogenic fungi, including *F. graminearum*, *M. oryzae*, and *Botrytis cinerea*, have shown that disruption of genes involved in carbohydrate metabolism, plant cell wall degradation, or sugar transport leads to reduced growth on specific carbon sources and attenuated virulence [39,40,41,42]. Similarly, in *V. dahliae*, enzymes involved in plant cell wall degradation and sugar metabolism have been reported to contribute to fungal development, microsclerotia formation, and pathogenicity [43]. Given that successful host colonization depends on efficient nutrient utilization, impaired carbon metabolism may contribute to the decreased pathogenicity of the Δ*VdPRMT1* mutant.

A notable finding of this study is the identification of VdLuc7 as a direct interacting partner of VdPRMT1. Based on the enrichment of RG/RGG motifs—common in PRMT substrates [8], we selected VDAG_05301, which contains seven such motifs, for validation. A specific interaction between VdPRMT1 and VDAG_05301 was confirmed, suggesting it may be a potential PRMT-associated protein. VDAG_05301 was identified as a member of the Luc7 protein family based on conserved domain analysis, so it was named VdLuc7. Luc7 proteins are conserved components of the U1 small nuclear ribonucleoprotein (U1 snRNP) complex and are involved in pre-mRNA processing [44,45]. The interaction between VdPRMT1 and VdLuc7 suggests a potential association between arginine methylation and RNA processing in *V. dahliae*. In addition, repeated failure to obtain VdLuc7 deletion mutants suggests that it may be important for fungal viability or growth under the tested conditions (Figure S5). Recent studies have also shown that LUC7 family proteins can influence RNA processing and cellular metabolism [46], supporting a potential functional association between VdPRMT1-mediated arginine methylation and RNA-related regulatory pathways in *V. dahliae*. Given that proper regulation of gene expression is essential for fungal infection and host colonization, the interaction between VdPRMT1 and VdLuc7 may be associated with the regulation of pathogenicity-related processes.

Finally, the reduced virulence observed in the Δ*VdPRMT1* mutant highlights the critical role of VdPRMT1 in fungal pathogenicity and suggests that it represents a potential molecular target for disease control. Although host-induced gene silencing (HIGS) has been widely used to control fungal pathogens by targeting essential genes [47], our results further emphasize the potential of targeting key regulatory factors involved in fungal growth and virulence [28]. Given its central role in regulating fungal development and pathogenicity, VdPRMT1 may represent a promising candidate for developing novel strategies to control Verticillium wilt. Future studies focusing on its downstream targets and regulatory mechanisms will further enhance our understanding of its biological functions.

## 5. Conclusions

Using genetic, transcriptomic, and protein interaction approaches, we demonstrate that VdPRMT1 is an important regulator of fungal growth, stress responses, carbon utilization, and pathogenicity in *V. dahliae*. The identification of VdLuc7 as an interacting partner further suggests that arginine methylation may be associated with RNA-related regulatory pathways. These findings highlight the importance of PRMT1-mediated regulation in fungal biology and provide new insights into the molecular basis of pathogenicity in plant pathogenic fungi.

## Data Availability

All the data are included in the manuscript and the Supporting Information. The RNA-seq datasets generated in this study have been deposited in the NCBI (https://www.ncbi.nlm.nih.gov/, 4 August 2026) database under accession number PRJNA1454220.

## Supplementary Materials

The following supporting information can be downloaded at: https://www.mdpi.com/article/10.3390/cells15151425/s1, Table S1. Primer sequences used in the experiment; Table S2. Yeast two-hybrid screening of VdPRMT1-interacting candidate proteins in *Verticillium dahlia*; Figure S1. Mapping of recombinant vectors used in the experiment; Figure S2. Construction principle and PCR identification of *VdPRMT1* knockout mutant; Figure S3. *VdPRMT1* functional complement-related PCR identification; Figure S4. Sporulation analysis and global transcriptomic changes in the Δ*VdPRMT1* mutant; Figure S5. Failed attempts to generate *VdLuc7* deletion mutants in *V. dahliae*.
