# Supplementary material for: VdPRMT1 Is Required for Fungal Growth, Metabolism, and Pathogenicity in Verticillium dahliae
Source: Cells. 2026 Aug 6;15(15):1425. doi: 10.3390/cells15151425 (PMC13464437; doi:10.3390/cells15151425)
Supplement: Supplementary file 1 [file cells-15-01425-s001.zip › cells-4401835-supplementary.pdf]

Supplemental information

Table S1. Primers sequences used in the study

Primer Name	sequence（5′-3′）
PRMT1-pCHG-UF	GGTTGAATTCACCAGAGCTCCGTCTCATGTAATTCCAAGG
PRMT1-pCHG-UR	CTCCTTCAATATCAGGTACCCAACAGTGTGATCGTGAAG
PRMT1-pCHG-DF	GATGCCGACCGGGAGGATCCGACCGTCTTCTACCTCAAG
PRMT1-pCHG-DR	GCCTGCAGGTCGACTCTAGACATACACAACCTCCAACAAG
PRMT1-pMC-F	GAATTCTGCCACCTTGGATCCATGGCTGCCGAGAGAATGG
PRMT1-pMC-R	GCCCTTGCTCACCATGTCGACGCACATCTTGTAGGTGGCGG
PRMT1-qPCR-F	CGGCACTGCCATTCTGTCC
PRMT1-qPCR-R	TGTCGACCTTGGGGAAGGG
ACTIN-qPCR-F	GGCTTCCTCAAGGTCGGCTATG
ACTIN-qPCR-R	GCTGCATGTCATCCCACTTCTTC
PRMT1-TRV2-F	TCTGTGAGTAAGGTTACCGAATTCGGAGCACTCTGAGCAGCACTAC
PRMT1-TRV2-R	ACGCGTGAGCTCGGTACCGGATCCAGGTGTCACGAGCGTAGAGGA
PRMT1-BD-F	ATGGCCATGGAGGCCGAATTCATGGCTGCCGAGAGAATGG
PRMT1-BD-R	CCGCTGCAGGTCGACGGATCCCTAGCACATCTTGTAGGTGGCG
VD05301-AD-F	GCCATGGAGGCCAGTGAATTCATGGCCGCCGAACAACGG
VD05301-AD-R	CAGCTCGAGCTCGATGGATCCTTACCAGCCGCCGCGATA
PRMT1-VN-F	GCCGGCGCCGGCGCCGAATTCATGGCTGCCGAGAGAATGG
PRMT1-VN-R	TCAGTAACGTTAAGTGAATTCCTAGCACATCTTGTAGGTGGCG
05301-VC-F	CTCCATCACATCACAGGATCCATGGCCGCCGAACAACGG
05301-VC-R	GCCGGCGCCGGCGCCGGATCCCCAGCCGCCGCGATATCC
HPT-F	AAGTTTCGACAGCGTCTCCCGACCTGA
HPT-R	CAGTCCTCCGGCCCAAAAGCATCAGCT
05301-N-F	ACGGGGGACGAGCTCATGGCCGCCGAACAACGGAA
05301-N-R	GTATGGGTAGTCGACCCAGCCGCCGCGATATCCAC
PRMT1-C-F	GACGAGCTCGGTACCATGGCTGCCGAGAGAATGG

PRMT1-C-R	CGAGATCTGGTCGACCTAGCACATCTTGTAGGTGGCG
LUC7-3-F	GAGATCTAGAGGATCCCCCGACTAGTGCAGCAACTTCAGAAACGGTG
LUC7-3-R	TACCGGGCCCCCCCTCGAGGTCGATGTTACTTGATGGTGTACAGG
LUC7-5-F	CGCTCTAGAACTAGTGGATCCCCGGGTGCGACTCCGAGGTGACAAAG
LUC7-5-R	CATCGATGGGCCCGCTGAGGACATCGTACACAGCCTCATAAG
08591-qPCR-F	CGGCGGCGGGCCCAACGA
08591-qPCR-R	TCGTTGTAGTAGTGGCTCGG
02580-qPCR-F	GCTACCAGCTAGACGACCGT
02580-qPCR-R	CTTCTTCGGCCTCCTTGTCG
00419-qPCR-F	GCTCGACATTGGCTTCTTCC
00419-qPCR-R	CAGGTTGTTGATCTCGGAGC
00420-qPCR-F	CCTAGCTGCCTTCTTCATCG
00420-qPCR-R	AAACGTAACCGCGGATCGAG
08124-qPCR-F	GGTCTGTGACGTCGACCTTC
08124-qPCR-R	ATGGTCGGGTACAGGTCCTG
07881-qPCR-F	CTGACGAGCTACCAGGACAC
07881-qPCR-R	CAATGTCGCAGCCCTCGATC
01866-qPCR-F	GAGCACCAGGCTCTTGTCAC
01866-qPCR-R	GATAGATGACGGTGCGGAGC
07316-qPCR-F	CTGATGCAGGGCCACTTCAC
07316-qPCR-R	TGTGACGATGGTCCAGTCGG
02013-qPCR-F	TCCAGGAGGGCATCTACGAC
02013-qPCR-R	ATGCGGTCGAACTGCAGCTG
T7 Primer	TAATACGACTCACTATAGGG
AD Colony PCR-R	GTGAACTTGCGGGGTTTTTCAGTATCTACGATT

Table S2. Yeast two-hybrid screening of VdPRMT1-interacting candidate proteins in *Verticillium dahliae*

Genbank	Candidate targets	Number of RG/RGG domains
VDAG_10312	iron sulfur assembly protein	1
VDAG_04315	proliferating cell nuclear antigen	0
VDAG_05365	ubiquitin-conjugating enzyme	0
VDAG_09413	LYR family protein	1
VDAG_02648	Synaptobrevin	2
VDAG_05286	hypothetical protein	0
VDAG_08094	hypothetical protein	5
VDAG_03021	septation protein SUN4	0
VDAG_03310	hypothetical protein	2
VDAG_10367	hypothetical protein	0
VDAG_06194	hypothetical protein	2
VDAG_07278	hypothetical protein	1
VDAG_09948	40S ribosomal protein S20	0
VDAG_06910	hsp70-like protein	1
VDAG_08390	lysine decarboxylase family	2
VDAG_07130	hypothetical protein	2
VDAG_05301	LUC7 protein	7
VDAG_01494	hypothetical protein	1
VDAG_05516	78 kDa glucose-regulated protein	1
VDAG_02507	60S ribosomal protein L14-B	1
VDAG_07229	hypothetical protein	1
VDAG_05786	60S ribosomal protein L10a	0
VDAG_04551	antigen 1	1
VDAG_05285	hypothetical protein	2
VDAG_08716	hypothetical protein	3
VDAG_10492	40S ribosomal protein S27a	1

VDAG_03288	TGF-beta-inducible nuclear protein	2
VDAG_10051	Tyrosinase	2
VDAG_03653	hypothetical protein	1
VDAG_08950	activating signal cointegrator 1 complex subunit 3	4
VDAG_06911	hypothetical protein	3
VDAG_08653	hypothetical protein	0
VDAG_00201	cellular nucleic acid-binding protein	0
VDAG_00452	PBSP domain-containing protein	1

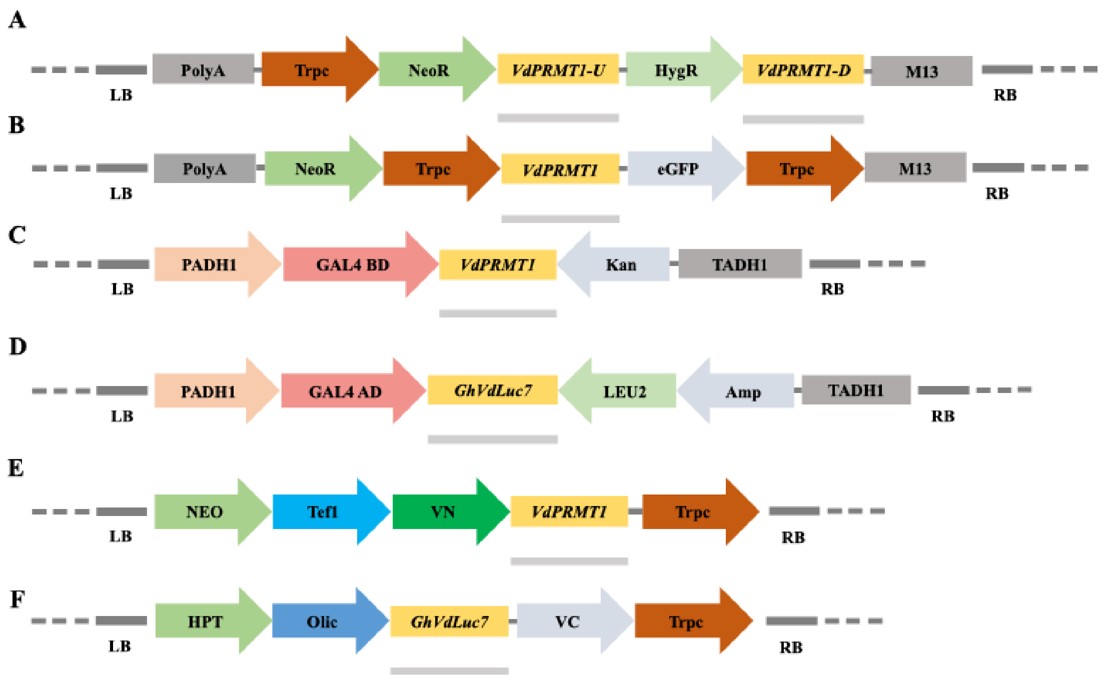

Figure S1. Mapping of recombinant vectors used in the experiment. (A) Knockdown vector pCHG-VdPRMT1: containing upstream and downstream homology arms of the VdPRMT1 gene (U, D) and the thiamphenicol resistance gene (Hyg); (B) Replacement vector pMC-VdPRMT1: contains the complete CDS sequence of VdPRMT1 and EGFP tag; (C) pGBKT7-VdPRMT1: the coding region of VdPRMT1 is fused to the BD domain; (D) pGADT7-VdLuc7: VdLuc7 coding region is fused with AD domain; (E) pB-NEO-VN-VdPRMT1: carries a fragment of VdPRMT1 fused to a VN tag; (F) pB-HPT-VC-VdLuc7: carries a fragment of VdPRMT1 fused to a VC tag.

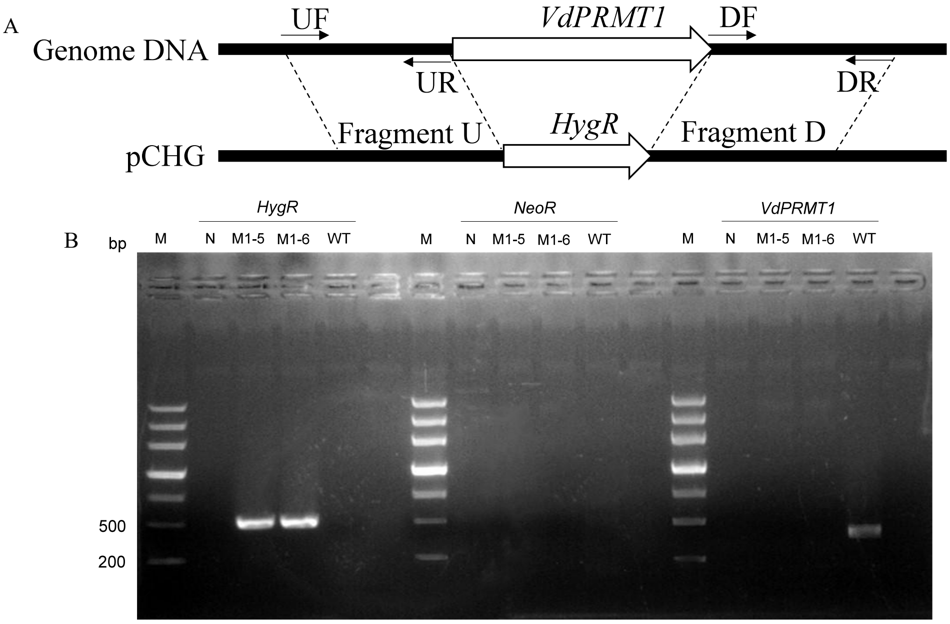

Figure S2. Construction principle and PCR identification of *VdPRMT1* knockout mutant. (A) The knockout principle diagram: through homologous recombination, the upstream homologous arm (Fragment U), hygromycin resistance gene (HygR), and downstream homologous arm (Fragment D) on the vector pCHG were used to replace the VdPRMT1 gene in genomic DNA; UF and UR are the amplification primers of the upstream homologous arm, and DF and DR are the amplification primers of the downstream homologous arm; (B) PCR identification results : M is the DNA molecular weight standard, N is the negative control, M1-5, M1-6 are VdPRMT1 knockout mutants, WT is wild type. The HygR primer only amplified specific bands in the mutant, the NeoR primer did not amplify (the vector pCHG contained NeoR, but was not integrated into the genome), and the VdPRMT1 primer only amplified bands in the wild type, indicating that the VdPRMT1 knockout mutant was successfully constructed.

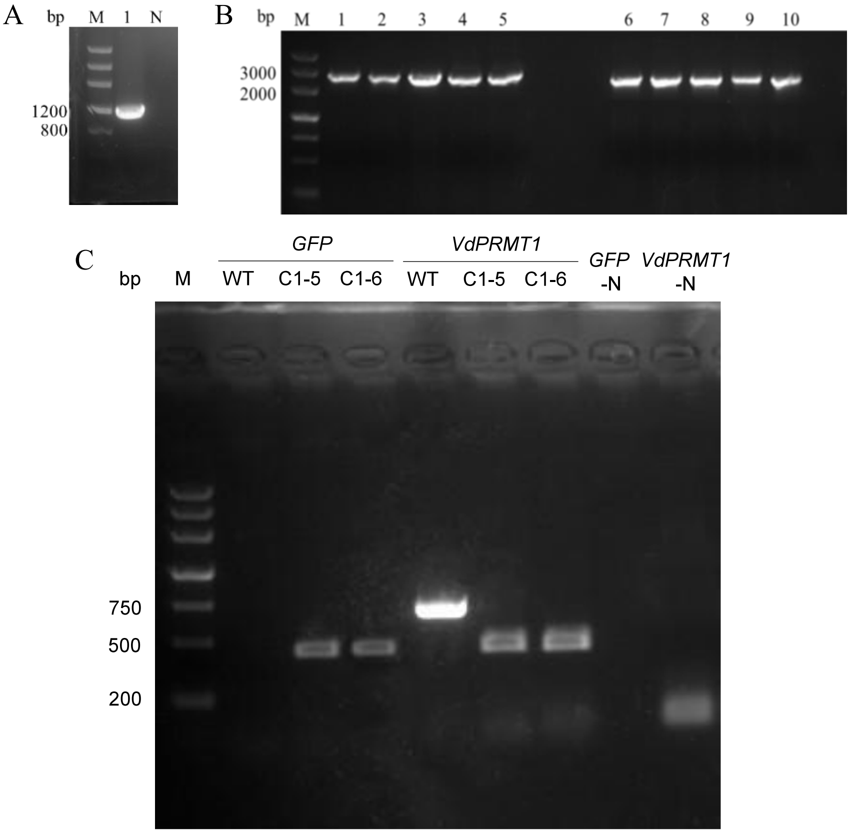

Figure S3. VdPRMT1 functional complement related PCR identification. (A) The PCR amplification results of VdPRMT1 CDS sequence (without stop codon), M was the DNA molecular weight standard, 1 was the amplification product, and N was the negative control; (B) The bacterial liquid PCR identification of the complement plasmid pMC-VdPRMT1 showed that M was the DNA molecular weight standard, and 1-10 were different monoclonal bacterial liquid PCR products; (C) PCR identification of VdPRMT1 functional complement. M is the DNA molecular weight standard. The amplification of GFP (C1-5, C1-6 are complement strains) and VdPRMT1 gene in wild type (WT) and complement strains was detected. GFP-N and VdPRMT1-N were negative controls.

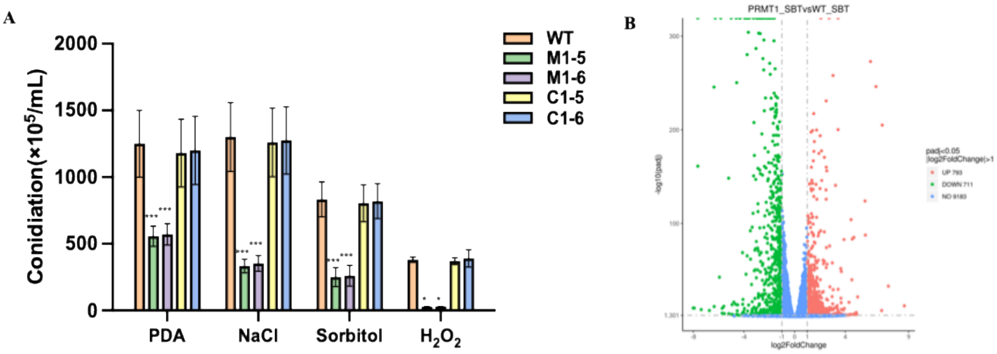

Figure S4. Sporulation analysis and global transcriptomic changes in the Δ *VdPRMT1* mutant. (A) Sporulation quantity of wild type (WT), VdPRMT1 knockout mutants (M1-5, M1-6), and complementation strains (C1-5, C1-6) cultured on PDA and different stress medium for 12 days. (B) Volcano plot showing differentially expressed genes between the wild-type strain and the Δ *VdPRMT1* mutant.

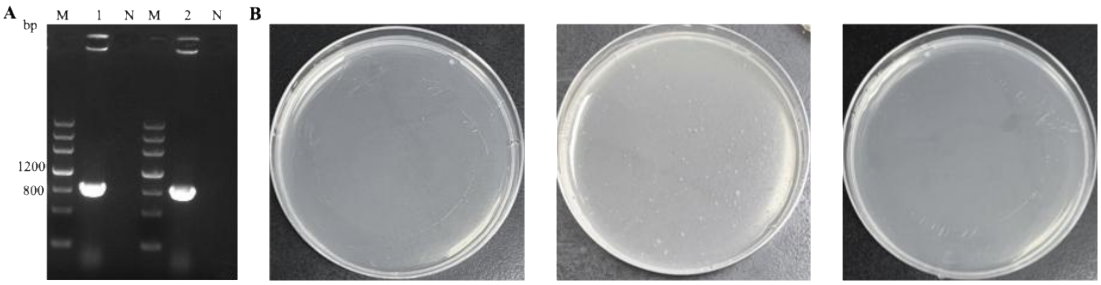

Figure S5. Failed attempts to generate VdLuc7 deletion mutants in *V. dahliae*. (A) PCR amplification of upstream and downstream fragments of VdLuc7. M was the DNA molecular weight standard; 1 was the upstream homologous arm product; 2 was the downstream homologous arm ; N was the negative control (B) Transformant colonies obtained on selective medium during attempts to generate VdLuc7 deletion mutants.
